# Prediction value study of breast cancer tumor infiltrating lymphocyte levels based on ultrasound imaging radiomics

**DOI:** 10.3389/fonc.2024.1411261

**Published:** 2024-06-06

**Authors:** Min Zhang, Xuanyu Li, Pin Zhou, Panpan Zhang, Gang Wang, Xianfang Lin

**Affiliations:** ^1^ Department of Ultrasound, Taizhou Hospital of Zhejiang Province Affiliated to Wenzhou Medical University, Taizhou, Zhejiang, China; ^2^ Department of Pathology, Taizhou Hospital of Zhejiang Province, Taizhou, Zhejiang, China; ^3^ Department of Ultrasound, Taizhou Hospital of Zhejiang Province, Taizhou, Zhejiang, China

**Keywords:** breast cancer, tumor-infiltrating lymphocytes, ultrasound, radiomics, predictive model

## Abstract

**Objective:**

Construct models based on grayscale ultrasound and radiomics and compare the efficacy of different models in preoperatively predicting the level of tumor-infiltrating lymphocytes in breast cancer.

**Materials and methods:**

This study retrospectively collected clinical data and preoperative ultrasound images from 185 breast cancer patients confirmed by surgical pathology. Patients were randomly divided into a training set (n=111) and a testing set (n=74) using a 6:4 ratio. Based on a 10% threshold for tumor-infiltrating lymphocytes (TIL) levels, patients were classified into low-level and high-level groups. Radiomic features were extracted and selected using the training set. The evaluation included assessing the relationship between TIL levels and both radiomic features and grayscale ultrasound features. Subsequently, grayscale ultrasound models, radiomic models, and nomograms combining radiomics score (Rad-score) and grayscale ultrasound features were established. The predictive performance of different models was evaluated through receiver operating characteristic (ROC) analysis. Calibration curves assessed the fit of the nomograms, and decision curve analysis (DCA) evaluated the clinical effectiveness of the models.

**Results:**

Univariate analyses and multivariate logistic regression analyses revealed that indistinct margin (P<0.001, Odds Ratio [OR]=0.214, 95% Confidence Interval [CI]: 0.103-1.026), posterior acoustic enhancement (P=0.027, OR=2.585, 95% CI: 1.116-5.987), and ipsilateral axillary lymph node enlargement (P=0.001, OR=4.214, 95% CI: 1.798-9.875) were independent predictive factors for high levels of TIL in breast cancer. In comparison to grayscale ultrasound model (Training set: Area under curve [AUC] 0.795; Testing set: AUC 0.720) and radiomics model (Training set: AUC 0.803; Testing set: AUC 0.759), the nomogram demonstrated superior discriminative ability on both the training (AUC 0.884) and testing (AUC 0.820) datasets. Calibration curves indicated high consistency between the nomogram model’s predicted probability of breast cancer TIL levels and the actual occurrence probability. DCA revealed that the radiomics model and the nomogram model achieved higher clinical net benefits compared to the grayscale ultrasound model.

**Conclusion:**

The nomogram based on preoperative ultrasound radiomics features exhibits robust predictive capacity for the non-invasive evaluation of breast cancer TIL levels, potentially providing a significant basis for individualized treatment decisions in breast cancer.

## 1 Introduction

The latest 2022 statistics reveal that there were 287,850 new breast cancer cases among American women, thereby positioning breast cancer as the most common malignancy among women. It is also a leading cause of cancer-related deaths in women (1).Thus, early diagnosis and selecting effective treatment strategies are imperative for patient survival and prognosis.

Tumor-Infiltrating Lymphocytes (TIL) are mononuclear cells prevalent in the tumor interior and its surrounding matrix. They influence tumor cell metabolism and local immune responses, playing a role in the body’s immune reactions. Many studies have found that when overall TIL levels or specific subgroups within the stroma increase, pre-existing anti-tumor responses are enhanced. Higher levels of TIL serve as prognostic factors for patients undergoing adjuvant chemotherapy and are also biomarkers for increased pathological response after neoadjuvant chemotherapy.

In a study of 2148 breast cancer patients, higher TIL levels in TNBC were associated with a 17% reduction in recurrence risk and a 27% reduction in mortality risk (2). Additionally, two multicenter randomized trials anthracycline-based adjuvant chemotherapy revealed that TNBC patients with high TIL levels had a 10-year overall survival rate of 89%, whereas those with low levels had a rate of 68%. For HER2-positive patients, the corresponding 10-year overall survival rates were 78% and 57%, respectively (3). In the GeparSixto trial, tumors with high TIL levels had a 59.9% pathological complete response rate, compared to 33.8% for tumors with low levels (4). Moreover, studies suggest that the effectiveness of immune checkpoint blockade (ICB) treatments correlates with the quality and extent of TIL reactions in the tumor microenvironment (5). In a phase I clinical study of metastatic TNBC patients treated with atezolizumab monotherapy, those with TIL > 10% had significantly better median overall survival than those with TIL < 10% (6).

Hence, identifying effective methods to evaluate TIL levels in breast cancer is essential, as it enables identification of patients more likely to respond to immune modulation and chemotherapy, thereby facilitating more precise personalized treatments and improving patient outcomes.

In 2014, the International Immuno-Oncology Biomarker Working Group on Breast Cancer issued the latest guidelines for TIL assessment, specifying the methodology for evaluating TIL in breast cancer pathology sections by calculating the percentage of TIL in the entire tumor stroma (7). However, pathology sections have their limitations; obtaining the full extent of TIL infiltration in the tumor requires excising the entire lesion, which is impractical for patients unable to undergo initial surgery. Moreover, due to tumor spatial heterogeneity, TIL levels based on tissue biopsies may not accurately reflect the entire tumor’s TIL infiltration (8). Research also indicates that cytotoxic chemotherapy induces dynamic changes in the tumor immune microenvironment, meaning TIL levels in the tumor stroma vary during chemotherapy, differing by molecular subtype and pathological response (9). Consequently, monitoring TIL levels during chemotherapy would necessitate multiple invasive biopsies, a challenging proposition in clinical practice. Thus, there is an urgent need for a more objective, reliable, and non-invasive method to assess preoperative TIL infiltration levels in breast cancer.

Presently, imaging methods such as molybdenum target mammography, ultrasound, and Magnetic Resonance Imaging (MRI) are instrumental in diagnosing and treating breast cancer, offering a holistic, non-invasive evaluation of the tumor’s characteristics. Traditional imaging, however, is limited to morphological diagnosis and does not provide the molecular and genetic information required for precision medicine. Recently, radiomics has emerged as a significant field. This computer-aided technique, introduced by Lambin et al (10) in 2012, entails the high-throughput extraction and quantitative analysis of numerous image features from medical images, particularly from the Region of Interest (ROI). This process yields key insights, including high-dimensional, quantifiable, and analyzable data regarding the tumor’s potential tissue characteristics, which are distinct from other data types like clinical, treatment-related, or genomic data (11, 12). It can be utilized either independently or in conjunction with demographic, histological, genomic, or proteomic data to tackle clinical challenges (13). Numerous studies have demonstrated the effectiveness of ultrasound-based radiomics in diagnosing and distinguishing breast diseases, as well as in evaluating immunohistopathological features, thus aiding in patient treatment decisions (14–16). While there have been smaller studies using molybdenum target mammography and MRI to assess the level of TIL in breast cancer (17–19), breast ultrasound, recognized for its safety, absence of radiation, non-invasiveness, and cost-effectiveness, is more readily accepted by patients and is a key imaging modality in breast cancer. However, there are no reports yet on employing ultrasound radiomics for assessing breast cancer TIL levels. Therefore, we posit that ultrasound radiomics can evaluate the TIL level in breast cancer patients, proposing the development of a novel ultrasound radiomics assessment system. This system aims to predict TIL levels in breast cancer, guiding the creation of more precise clinical diagnosis and treatment strategies and improving the prognosis for breast cancer patients.

## 2 Materials and methods

### 2.1 Patients

**Figure 1:**
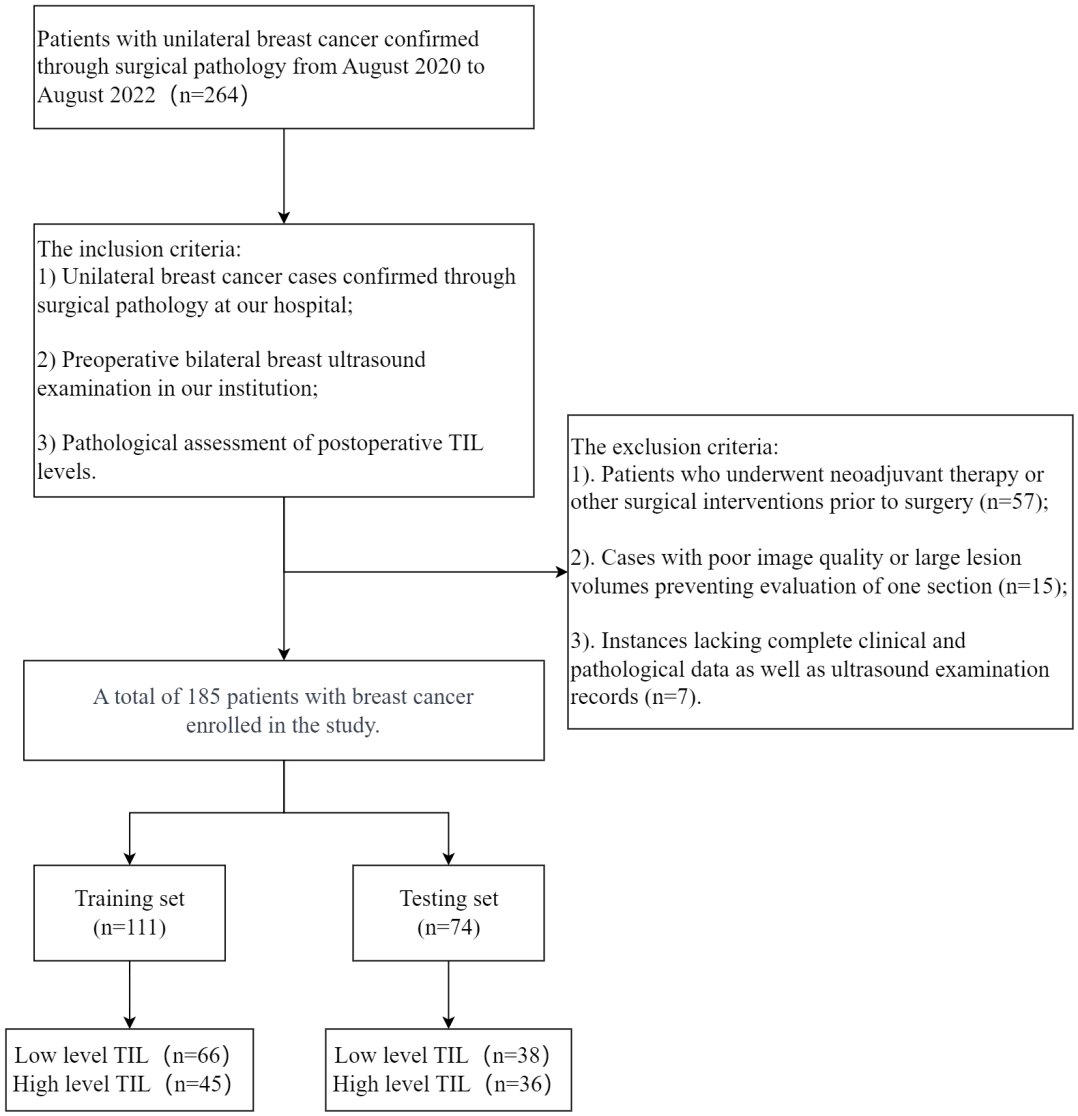
A flowchart illustrating the patient inclusion process for this study.

We retrospectively collected data from 185 female (mean age 54 ± 10) with 194 breast cancer lesions diagnosed in our hospital between August 2020 and August 2022. For cases with multiple lesions, only the largest lesion was selected for radiological and histopathological evaluation. This study is a single-center retrospective study approved by Taizhou Hospital of Zhejiang Province ethics committee, with the requirement for informed consent waived. Patients were randomly assigned to training and testing sets in a ratio of 6:4. Inclusion criteria were as follows: 1) Unilateral breast cancer confirmed by surgical pathology at our hospital; 2) Preoperative bilateral breast ultrasound examination was mandatory; 3) Pathological evaluation of TIL levels after surgery. Exclusion criteria included: 1) Patients who received neoadjuvant treatment or other surgical interventions before surgery; 2) Poor image quality or large lesion volume resulting in the inability to assess a specific section; 3) Lack of complete clinical pathological data and ultrasound examination data. The exclusion and inclusion criteria are illustrated in **Figure 1**.

### 2.2 Clinical and pathological characteristics

Record clinical and pathological data of the tumor, including age, histopathological subtype, molecular expression status, molecular subtyping, histological grade, and ipsilateral axillary lymph node metastasis.

Histological subtypes include invasive ductal carcinoma (IDC), ductal carcinoma *in situ* (DCIS), and others (such as invasive lobular carcinoma, lobular carcinoma in situ, etc.).

Based on the immunohistochemical results of estrogen receptor (ER), progesterone receptor (PR), and HER2-amplified, patients are categorized into the following four types:

(1) Luminal A: ER/PR (+), HER-2(-), and Ki-67<14%.
(2) Luminal B: ER/PR (+), HER-2(-), and Ki-67≥14%; or ER/PR (+) and HER-2(+).
(3) TNBC: ER (-), PR (-) and HER-2(-).
(4) HER-2 -amplified: ER (-), PR (-) and HER-2(+).

### 2.3 Histopathological evaluation of TIL

In line with the 2014 recommendations from the International Immuno-Oncology Biomarker Working Group on Breast Cancer (7), We primarily assessed and analyzed TIL within the stromal area. The TIL score is defined as the percentage of TIL the entire stromal area. This assessment is conducted on breast cancer pathological sections. TIL should be evaluated within the invasive tumor margin (IM), which is defined as the region centered on the boundary between normal tissue and cancer nests, with a range of 1mm. TIL assessment requires the exclusion of TIL within tumor cells, *in situ* carcinoma, surrounding normal lobules, tissue damage, necrosis, fibrotic areas, and tertiary lymphoid structures. Only mononuclear cells (lymphocytes and plasma cells) are assessed. Different areas of the tumor are selected for TIL assessment to obtain the average TIL count, rather than focusing only on hotspots. In this study, TIL levels were categorized as low (<10%) and high (≥10%). Two pathologists with five years or more of work experience evaluated the lesions without knowing any clinical data or pathological results. They categorized all specimens into low TIL level group and high TIL level group.

### 2.4 Ultrasound image acquisition and feature analysis

A Samsung XW80A color Doppler ultrasound machine with a real-time linear array high-frequency probe (frequency range: 7.5-15.0 MHz) was utilized for multi-angle and multi-sectional scanning of the lesion areas without any marking. Simultaneously, color Doppler was employed to examine the vascularization of the lesions. Two diagnostic physicians with more than five years of experience in superficial ultrasound reviewed the images. The physicians were unaware of the patients’ information. Based on the ACR 2013 edition Breast Imaging Reporting and Data System (BIRADS) diagnostic criteria, the image features were evaluated. In case of disagreements, consensus was reached through discussion, and the evaluation results were recorded. The assessed features included the maximum diameter of the mass, growth direction (parallel or non-parallel), morphology (regular or irregular), margins (indistinct, spiculated, angular, micro-lobulated), internal echogenicity (low echogenicity, cystic-solid echogenicity, heterogeneous echogenicity), microcalcifications (present or absent), posterior echoes (no apparent changes, enhancement, attenuation, mixed changes), color doppler flow imaging (according to the Adler method: Grade 0, no blood flow signal detected within the mass; Grade 1, minimal blood flow, with 1-2 punctate or rod-like tumor vessels visible; Grade 2, moderate blood flow, with 3-4 punctate vessels or a longer vessel entering the lesion, with its length approaching or exceeding the radius of the mass; Grade 3, abundant blood flow, with more than 5 punctate vessels or 2 longer vessels visible). Additionally, the status of ipsilateral axillary lymph node enlargement (cortical thickening, length-to-width ratio < 2.0 defined as lymph node enlargement) was assessed.

### 2.5 Grayscale ultrasound model construction

In conducting univariate analysis to compare grayscale ultrasound differences among patients in various TIL groups, features exhibiting significance at P < 0.05 are identified as risk factors for evaluating high TIL levels. Subsequently, these significant features are incorporated into multivariate logistic regression analysis. Grayscale ultrasound features with a significance level of P < 0.05 in the multivariate analysis are chosen to formulate a grayscale ultrasound model. Odds ratios (OR) along with 95% confidence intervals (CI) are then computed to evaluate the relative risk associated with each predictive factor.

### 2.6 Radiomics analysis and model construction

#### 2.6.1 ROI segmentation and feature extraction

**Figure 2:**
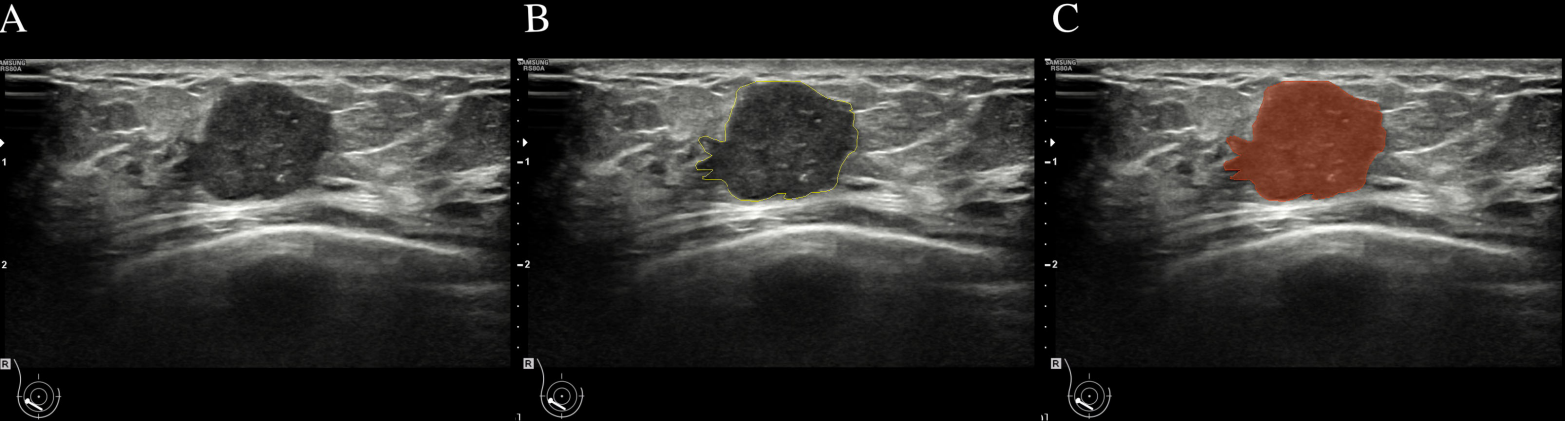
Breast mass ultrasound image region of interest (ROI) delineation schematic. **(A)**:Two-dimensional ultrasound image of a breast mass. **(B)**:Delineate the boundaries of ROI **(C)**:the mask image segmented from **(A)**.

Ultrasound images of the lesion’s maximum diameter were imported into the 3D Slicer software (version Slicer-5.0.2). An ultrasound doctor (A) with 5 years or more of ultrasound diagnostic experience manually delineated the Region of Interest (ROI) along the tumor contour on the grayscale ultrasound image (**Figure 2**). Feature extraction was conducted using the pyradiomics software package based on the Python. The extracted features included several categories: first-order features, shape features (2D), gray-level co-occurrence matrix features (GLCM), gray-level size zone matrix features (GLSZM), gray-level run length matrix features (GLRLM), neighboring gray tone difference matrix features (NGTDM), gray-level dependence matrix features (GLDM), and wavelet transform features.

#### 2.6.2 Intra-observer and inter-observer agreement for defining the ROI

We randomly selected 20 images, and the same ultrasound doctor(A) repeated ROI segmentation after two weeks to analyze intra-observer repeatability. Another ultrasound doctor (B) performed ROI segmentation independently during the same period to analyze inter-observer repeatability. One-way analysis of variance (ANOVA) was used to calculate the Intraclass Correlation Coefficient (ICC) to quantify the consistency within and between observers. The ICC values range from 0 to 1, with values closer to 1 indicating higher consistency. We selected radiomics features with ICC values all greater than 0.75.

#### 2.6.3 Feature selection and model development

The features of both the training and testing sets were standardized using Z-Score. Subsequently, dimensionality reduction and elimination of redundant features were performed on the training set through univariate analysis and the Least Absolute Shrinkage and Selection Operator (LASSO) regression algorithm. This process aimed to identify the most relevant features. For the univariate analysis, only features with P < 0.05 were retained. As for the LASSO method, feature selection was carried out through ten-fold cross-validation to obtain the final radiomics features and their coefficients. The selected features were weighted based on their coefficients to calculate the Rad-score for each patient. Finally, a multivariate logistic regression analysis was employed to construct the radiomics model.

### 2.7 Construction of the nomogram model and evaluation of models

A multivariate logistic regression analysis was employed to jointly establish the nomogram model, incorporating predictive factors from grayscale ultrasound features with the Rad-score. ROC curves were utilized to assess the performance of different models, calculating AUC values, accuracy, sensitivity, specificity, Positive Predictive Value (PPV), and Negative Predictive Value (NPV). The Delong test was applied for pairwise comparisons of ROC curves. Additionally, calibration curves were used to evaluate the consistency between clinical application and the actual probability versus predicted probability. The goodness-of-fit of the model was assessed through the Hosmer-Lemeshow test. Finally, Decision Curve Analysis (DCA) was conducted to evaluate the clinical application value of the nomogram

### 2.8 Statistical analysis methodology

The statistical analyses were conducted using IBM SPSS 25.0 and R software (version 3.6.0; available at http://www.Rproject.org.). For the analysis of clinical data, qualitative variables were compared using the Chi-square test or Fisher’s exact test, while quantitative variables were analyzed using the independent sample T-test. The R software facilitated various aspects of the analysis: the “glmnet” package was employed for LASSO analysis, with 10-fold cross-validation to enhance model optimization; the “psych” package was utilized for ICC test; ROC curves were generated using the “pROC” package; and the “rms” package was applied for conducting multivariate logistic regression, creating nomograms, and plotting calibration curves. Additionally, Decision Curve Analysis (DCA) curves were created using the “rmda” package, aiding in the evaluation of the model’s clinical applicability.

## 3 Results

### 3.1 Clinical and pathological characteristics

This study included a total of 185 breast cancer patients, with 101 cases (56.2%) in the low-level group and 84 cases (43.8%) in the high-level group. Significant differences between the two groups were observed in the expression of ER (P=0.007) and PR (P=0.024), ki67 levels (P=0.001), molecular subtypes (P<0.001), and histological grade (P=0.002). No statistical differences were found between the high and low-level groups in terms of age (P=0.746), axillary lymph node metastasis (P=0.693), HER2 expression (P=0.455), and histopathological subtypes (P=0.252). **Table 1** presents the clinical and pathological characteristics of the patients.

**Table 1:** Clinical and pathological characteristics of patients with different TIL levels.

Variable	All(n=185)	Low level(n=104)	High level(n=81)	P-value
Age, Mean ± SD (years)	53.70 ± 9.62	53.50 ± 9.36	53.96 ± 10.00	0.746
Axillary lymph node metastasis				0.693
Negative	116 (62.7)	67 (64.6)	49 (60.5)	
Positive	69 (37.3)	37 (35.6)	32 (39.5)	
ER status				0.007*
Negative	47 (25.4)	18 (17.3)	29 (35.8)	
Positive	138 (74.6)	86 (82.7)	52 (64.2)	
PR status				0.024*
Negative	56 (30.3)	24 (23.1)	32 (39.5)	
Positive	129 (69.7)	80 (76.9)	49 (60.5)	
HER2 status				0.455
Negative	143 (77.3)	83 (79.8)	60 (74.1)	
Positive	42 (22.7)	21 (20.2)	21 (25.9)	
Ki67 status				0.001*
Low (<14%)	38 (20.5)	31 (29.8)	7 (8.6)	
High (≥14%)	147 (79.5)	73 (70.2)	74 (91.4)	
Molecular subtype				<0.001*
Luminal A	37 (20.0)	30 (28.8)	7 (8.6)	
Luminal B	102 (55.1)	56 (53.8)	46 (56.8)	
TNBC	24 (13.0)	5 (4.8)	19 (23.5)	
HER2-amplified	22 (11.9)	13 (12.5)	9 (11.1)	
Histological grade				0.002*
I	19 (10.3)	16 (15.4)	3 (3.7)	
II	98 (53.0)	61 (58.7)	37 (45.7)	
III	55 (29.7)	22 (21.2)	33 (40.7)	
Null	13 (7.0)	5 (4.8)	8 (9.9)	
Histological subtype				0.252
IDC	175 (94.6)	100 (96.2)	75 (92.6)	
DICS	5 (2.7)	3 (2.9)	2 (2.5)	
Others	5 (2.7)	1 (1.0)	4 (4.9)	

*P<0.05.

### 3.2 Grayscale ultrasound feature analysis and model construction

Results from univariate analysis revealed statistically significant differences in tumor morphology (P=0.001), presence of spiculated margins (P=0.03), margin clarity (P<0.001), posterior enhancement (P<0.001), and ipsilateral axillary lymph node enlargement (P=0.007) among TIL subgroups in ultrasound images, as shown in **Table 2**. Multivariate logistic regression analysis demonstrated that blurry margins (P<0.001, OR=0.214, 95% CI: 0.103-1.026), posterior enhancement (P=0.027, OR=2.585, 95% CI: 1.116-5.987), and ipsilateral axillary lymph node enlargement (P=0.001, OR=4.214, 95% CI: 1.798-9.875) were independent predictors of high TIL levels (**Table 3**). The grayscale ultrasound model constructed based on these independent predictors showed an AUC, accuracy, sensitivity, specificity, PPV, NPV, and 95% CI of 0.795, 0.757, 0.644, 0.833, 0.725, 0.775, and 0.705-0.885 in the training set, and 0.720, 0.689, 0.528, 0.840, 0.760, 0.653, and 0.604-0.837 in the testing set (**Table 4**).

**Table 2:** Grayscale ultrasound features of breast cancer patients with different TIL levels.

Variable	Low level(N=104)	High level(N=81)	P value
Shape			0.001*
Regular	4(3.8)	16(19.8)	
Irregular	100(96.2)	65(80.2)	
Growth direction			0.666
Parallel	65(62.5)	54(66.7)	
Non-parallel	39(37.5)	27(33.3)	
Margin			
Spiculated	64(61.5)	36(44.4)	0.030*
Angular	77(74.0)	57(70.4)	0.698
Indistinct	75(72.1)	34(42.0)	<0.001*
Micro-lobulated	16(15.4)	19(23.5)	0.230
Echo pattern (%)			0.302
Hypoechoic	96(92.3)	69(85.2)	
Heterogeneous	2(1.9)	3(3.7)	
Cystic-solid	6(5.8)	9(11.1)	
Posterior features (%)			<0.001*
No posterior features	45(43.3)	26(32.1)	
Enhancement	19(18.3)	31(38.3)	
Shadowing	34(32.7)	10(12.3)	
Combined pattern	6(5.8)	14(17.3)	
Microcalcification			1
Yes	64(61.5)	49(60.5)	
No	40(38.5)	32(39.5)	
Ipsilateral axillary lymph node enlargement			0.007*
Yes	15(14.4)	26(32.1)	
No	86(85.6)	55(67.9)	
Adler classification			0.847
Grade0-1	9(11.5)	14(13.6)	
Grade2-3	92(88.5)	70(86.4)	

*P<0.05.

**Table 3:** Multivariate logistic regression analysis of grayscale ultrasound features in nreast cancer patients with different TIL levels.

Variable	Multivariable Logistic Regression Analysis	
	OR (95%CI)	P-value
Shape	0.379(0.103,1.398)	0.145
Spiculated	0.494(0.237,1.026)	0.059
Indistinct	0.214(0.103,0.446)	<0.001*
Posterior features		0.006*
Enhancement	2.585(1.116,5.987)	0.027*
Shadowing	0.543(0.209,1.408)	0.209
Combined pattern	3.019(0.934,9.752)	0.065
Ipsilateral axillary lymph node enlargement	4.214(1.798,9.875)	0.001*

*P<0.05.

**Table 4:** Predictive performance of different models in the training and testing sets.

Models	AUC	95%CI	ACC	SEN	SPE	PPV	NPV
Training set (N=111)							
US	0.795	0.705-0.885	0.757	0.644	0.833	0.725	0.775
Radiomics	0.803	0.719-0.887	0.730	0.621	0.889	0.615	0.891
Nomogram	0.884	0.820-0.949	0.829	0.778	0.864	0.795	0.851
Testing set (N = 74)							
US	0.720	0.604-0.837	0.689	0.528	0.842	0.760	0.653
Radiomics	0.759	0.647-0.871	0.743	0.639	0.842	0.793	0.711
Nomogram	0.820	0.727-0.914	0.757	0.694	0.816	0.781	0.738

### 3.3 Radiomics feature selection and model construction

**Figure 3:**
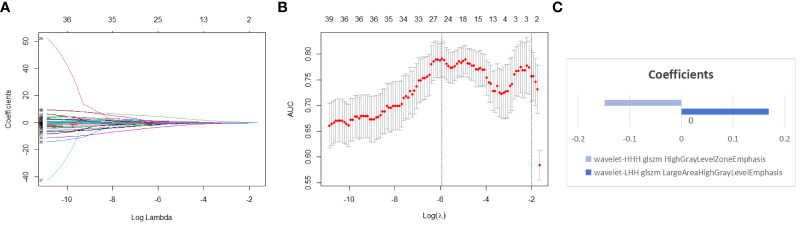
Using lasso regression model to select radiomics features **(A)** shows the variation of coefficients with the Log Lambda (Log λ) value. **(B)** depicts the selection of the most valuable features through lasso regression coupled with ten-fold cross-validation; the x-axis represents the Log (λ) value, with two vertical lines indicating the number of features at the optimal Log (λ) value and the minimum number of independent variables in the model with the best performance. **(C)** the two non-zero coefficient features of the simplest model we selected, along with the coefficient for each feature.

A total of 837 radiomics features were extracted from the original ultrasound images. After evaluating the consistency within and between observers, 664 features with an ICC greater than 0.75 were retained. Following one-way analysis of variance to eliminate features with low relevance, 42 features were retained. These features had ICC distributions intra-observers ranging from 0.831 to 0.997 and inter-observers ranging from 0.800 to 0.960. Further, LASSO regression analysis with ten-fold cross-validation identified two optimal features along with their respective coefficients (**Figures 3A–C**): “wavelet-LHH GLSZM Large Area High Gray Level Emphasis” and “wavelet-HHH GLSZM High Gray Level Zone Emphasis.” Rad-scores were calculated for each patient, and a multivariate logistic regression radiomics model was established. In the training set, the model exhibited an AUC, accuracy, sensitivity, specificity, PPV, NPV, and 95% CI of 0.803, 0.730, 0.621, 0.889, 0.615, 0.891, and 0.719-0.887, respectively. In the testing set, these values were 0.759, 0.743, 0.639, 0.842, 0.793, 0.711, and 0.647-0.871 (**Table 4**).

### 3.4 Construction of the nomogram model and evaluation of models

**Figure 4:**
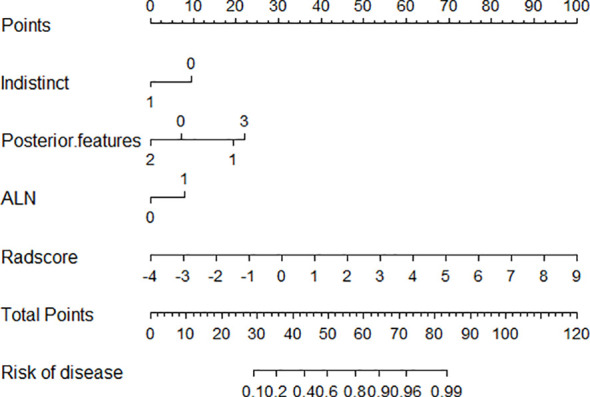
Nomogram model combining grayscale ultrasound features with Rad-score. Indistinct 0:no,1:yes. Posterior feature:0:No .posterior features,1:Enhancement,2: Shadowing,3:Combined pattern ALN represent Ipsilateral axillary lymph node enlargement,0:no,1:yes.

**Figure 5:**
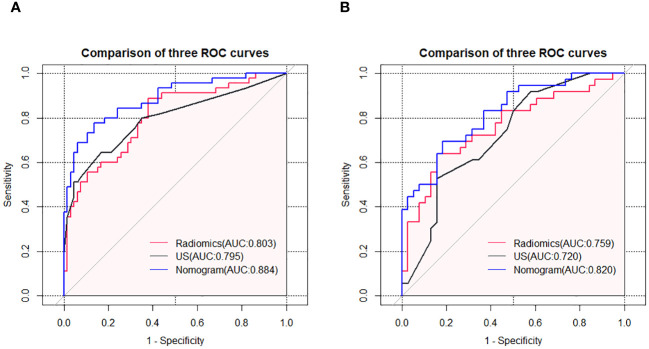
ROC curves of the grayscale ultrasound model, radiomics model, and nomogram model in the training set **(A)** and testing set **(B)**.

**Figure 6:**
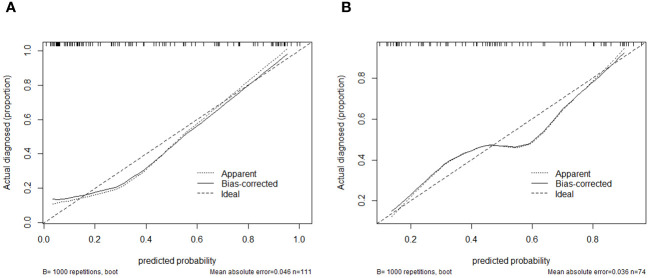
Calibration curves of the Nomogram Model in the training set **(A)** and testing set **(B)**.

**Figure 7:**
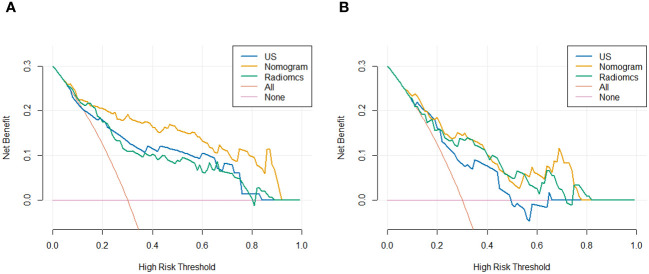
DCA for predicting breast cancer TIL levels by different models in the training set **(A)** and testing set **(B)**.

Using logistic regression, a radiomics nomogram model was constructed by combining Rad-score with grayscale ultrasound predictors (**Figure 4**). In the training set, the nomogram demonstrated an AUC of 0.884, accuracy of 0.829, sensitivity of 0.778, specificity of 0.864, PPV of 0.795, NPV of 0.851, and a 95% confidence interval of 0.820-0.949. In the testing set, these values were 0.820, 0.757, 0.694, 0.816, 0.781, 0.738, and 0.727-0.914, respectively. The ROC curves for all models are shown in **Figures 5A, B**. Delong’s test (**Table 5**) indicates that the nomogram model performs significantly better than the grayscale ultrasound model in both the training set (P=0.020) and testing set (P=0.024). In the training set, the nomogram model outperforms the radiomics model (P=0.005), but in the testing set, there is no significant statistical difference compared to the radiomics model (P=0.630). The radiomics model shows no significant statistical difference in performance compared to the grayscale ultrasound model in both the training set (P=0.891) and testing set (P=0.136). The calibration curves for the training set and testing set (**Figures 6A, B**), assessed using the Hosmer-Lemeshow test, showed no significant differences in both the training set (P=0.340) and testing set (P=0.147). This suggests good consistency between the actual and predicted risks of TIL levels. Decision Curve Analysis (DCA) (**Figures 7A, B**) demonstrated the clinical utility of the nomogram model. The results indicate that the combined radiomics nomogram model performs better in evaluating breast cancer TIL levels compared to the grayscale ultrasound model. The threshold probabilities in the training set and testing set range between 0.20-0.92 and 0.20-0.78, respectively.

**Table 5:** Comparison of ROC curve performance for different models in the training and testing sets.

Delong test	AUC of training set	P-value	AUC of testing set	P-value
Nomogram vs US	0.884 vs 0.795	0.020*	0.820 vs 0.720	0.025*
Nomogram vs Radiomics	0.884 vs 0.803	0.005*	0.820 vs 0.759	0.630
Radiomics vs US	0.803 vs 0.795	0.891	0.759 vs 0.720	0.136

*P<0.05.

## 4 Discussion

Recent research increasingly supports that TIL are crucial biomarkers for predicting the effectiveness of immunotherapy and neoadjuvant chemotherapy in breast cancer (3, 20–22).Given the complexity of postoperative pathological evaluations and the limitation of preoperative biopsies in fully representing the TIL level across the entire tumor, a non-invasive and practical method for assessing TIL levels is vital for tailored treatment strategies. Our study discovered that the nomogram, integrating optimal grayscale ultrasound and radiomics features and based on preoperative ultrasound radiomics, excels in evaluating TIL levels.

Earlier studies suggested a correlation between breast cancer TIL levels and ultrasound imaging characteristics (23), though these findings were not definitive. Our results indicate that tumors with elevated TIL levels are more likely to exhibit distinct margins and intensified posterior echoes on ultrasound, aligning with studies by Jia (24), Candelaria (25). The enhancement or attenuation of posterior echoes is associated with the tumor’s internal composition. It’s posited that increased TIL levels reflect a rise in water-soluble components and a reduction in collagen fibers within the tumor stroma. Additionally, tumor margins might influence, or be influenced by, the tumor microenvironment, potentially altering the tumor’s perimeter and interior. Hence, the intricate molecular and biological mechanisms governing the interplay between the tumor and its surrounding stroma warrant further investigation.

Celebi and colleagues (26) also identified variations in the appearance of tumor margins across different levels of TIL. They proposed that tumors with higher TIL levels tend to have larger volumes and more heterogeneous internal echoes. However, our study did not observe significant statistical differences in these aspects. Furthermore, MRI-based studies have also shown divergent outcomes regarding the correlation between tumor size and TIL levels (17, 27). The variance in findings related to tumor size may stem from different measurement approaches, such as assessments based on pathological specimens, the largest ultrasound cross-section, and the largest MRI cross-section. As for internal echoes, Fukui et al. in their sequential studies noted that higher TIL levels correlate with lower internal echo intensities (28, 29). In contrast, research focusing on TNBC revealed that tumors with high TIL levels display complex cystic and solid echo patterns (25). These variations might be attributed to the different classification systems used by Fukui’s team, such as the Japanese Breast and Thyroid Ultrasound Society, as well as differences in TIL level classification methods.

Our study also observed that tumors with higher levels of TIL were more prone to associated sentinel lymph node enlargement, aligning with findings from Chen et al. in MRI-based studies (30). Contrarily, Takada et al. (31) reported that TIL density in patients with lymph node metastasis was notably lower than in those without, focusing exclusively on T1 stage breast cancer patients, which may account for the disparities in our findings. Additionally, our investigation was limited to the morphological study of lymph nodes, necessitating larger sample sizes for more robust confirmation. Generally, grayscale ultrasound features correlate with TIL levels, yet they are operator-dependent and exhibit low reproducibility.

In our study, based on grayscale ultrasound images, we obtained a total of 837 rich quantitative features. Eventually, we narrowed down our selection to 2 wavelet transform features. Wavelet transform decomposes a signal into different frequency bands of wavelet coefficients, which can be utilized to analyze signal characteristics such as edges, textures, etc. The feature “Wavelet-LHH GLSZM Large Area High Gray Level Emphasis” quantifies large-sized, high-gray-level regions in the image, emphasizing their contribution to the overall texture. On the other hand, the feature “Wavelet-HHH GLSZM High Gray Level Zone Emphasis” quantifies regions with high gray levels in the image, highlighting their importance in the overall image. While the application of radiomics significantly mitigated the drawback of low repeatability in the original grayscale ultrasound images, our study results indicate that the incorporation of radiomic features significantly improved the predictive performance of the model (training set AUC=0.884, testing set AUC=0.820).

Jia et al.’s model, based on conventional ultrasound and contrast-enhanced ultrasound features, yielded an AUC of 0.790 in predicting TIL levels, further underscoring the superior predictive accuracy of ultrasound-based radiomics (24). Another analogous study reported a higher performance for their TIL-US scoring model (AUC=0.88) (29). However, this model involved subjective scoring of ultrasound features based on logistic regression outcomes and lacked validation in a new dataset, thus necessitating further verification of its generalizability.

Our work was contrasted with analogous radiomics studies based on mammography and MRI, as reported by Yu (19) and Xu et al (27). Their models achieved AUC values of 0.790 and 0.800, respectively, in the testing set, signifying moderate efficacy. Our study, with the nomogram model, demonstrated a slightly superior performance (AUC=0.820), albeit somewhat below that of the MRI-Dynamic Contrast-Enhanced (DCE) radiomics nomogram (AUC=0.840) (17).Despite this, the potential of ultrasound in accurately predicting TIL levels in breast cancer remains significant. Moreover, these studies, utilizing different imaging modalities, extracted diverse sets of radiomics features, and their approaches to delineating Regions of Interest (ROIs) also varied. Thus, future research will benefit from standardized feature extraction protocols to attain more consistent and enhanced outcomes.

This study has several limitations. Firstly, the sample size is relatively small, and it is a single-center retrospective study. Further research involving multiple centers and a larger number of patients is necessary. Secondly, our radiomic classifier was calculated using an ROI drawn only on the single largest slice in two-dimensional breast ultrasound images, which may introduce selection bias. However, breast ultrasound remains the most common method for breast cancer screening and diagnosis. Our study’s goal was to examine the predictive correlation between ultrasound presentations in breast cancer patients and their TIL levels. Should the radiomics predictive model, predicated on breast ultrasound imagery, prove effective in evaluating TIL levels, it could furnish radiologists and clinicians with invaluable insights, thereby enhancing clinical decision-making for breast cancer patients.

## 5 Conclusion

Radiomics introduces a novel approach for gleaning critical data from ultrasound images. The synergy of radiomics features with grayscale ultrasound characteristics has shown promising potential in accurately predicting the levels of TIL in breast cancer patients, thus facilitating clinical treatment strategies. The quantitative nomogram predictive model, anchored in the Rad-score and incorporating grayscale ultrasound features, emerges as a pivotal tool for the preoperative determination of TIL levels, potentially enriching clinical insights and decision-making in breast cancer management.

## Data availability statement

The raw data supporting the conclusions of this article will be made available by the authors, without undue reservation.

## Ethics statement

The studies involving humans were approved by Medical Ethics Committee of Taizhou Hospital of Zhejiang Province. The studies were conducted in accordance with the local legislation and institutional requirements. Written informed consent for participation was not required from the participants or the participants’ legal guardians/next of kin in accordance with the national legislation and institutional requirements.

## Author contributions

MZ: Conceptualization, Data curation, Formal analysis, Investigation, Methodology, Writing – original draft, Writing – review & editing, Software. XYL: Conceptualization, Data curation, Formal analysis, Investigation, Methodology, Writing – original draft, Writing – review & editing, Software. PZ: Data curation, Formal analysis, Investigation, Writing – original draft. PPZ: Data curation, Formal analysis, Investigation, Writing – original draft. GW: Data curation, Formal analysis, Investigation, Writing – original draft. XFL: Funding acquisition, Project administration, Resources, Supervision, Visualization, Writing – review & editing.
